# Dissociation Line and Driving Force for Nucleation of the Multiple Occupied Hydrogen Hydrate from Computer Simulation

**DOI:** 10.1021/acs.energyfuels.5c01012

**Published:** 2025-07-01

**Authors:** Miguel J. Torrejón, Samuel Blazquez, Jesús Algaba, Maria M. Conde, Felipe J. Blas

**Affiliations:** † Laboratorio de Simulación Molecular y Química Computacional, CIQSO-Centro de Investigación en Química Sostenible and Departamento de Ciencias Integradas, Universidad de Huelva, 21006 Huelva, Spain; ‡ Departamento de Química Física I, Fac. Ciencias Químicas, 16734 Universidad Complutense de Madrid, 28040 Madrid, Spain; § Departamento de Ingeniería Química Industrial y del Medio Ambiente, Escuela Técnica Superior de Ingenieros Industriales, 16743 Universidad Politécnica de Madrid, 28006 Madrid, Spain

## Abstract

In this work, we determine the dissociation temperature of hydrogen (H_2_) hydrate by computer simulation using two different methods. In both cases, the molecules of water and H_2_ are modeled using the TIP4P/Ice and a modified version of the Silvera and Goldman models, respectively, and the Berthelot combining rule for the cross water–H_2_ interactions has been modified. The first method used in this work is the solubility method, which consists of determining the solubility of H_2_ in an aqueous phase when in contact with the H_2_ hydrate (H–L_w_) phase and when in contact with the pure H_2_ phase (L_w_–L_H_2__) at different temperatures. At a given pressure value, both solubility curves intersect at the temperature (*T*_3_) at which the three phases coexist in equilibrium. Following this approach, we determine the dissociation temperature of H_2_ hydrate at 185 MPa finding a good agreement with the data previously reported in the literature. We also analyze the effect of the multiple occupancy of the D, or small, and H, or large, cages of the sII hydrate structure. We conclude that the *T*_3_ value is barely affected by the occupancy of the H_2_ hydrate at 185 MPa. From the analysis of the solubility curves and performing extra bulk simulations of the three phases involved in the equilibrium, we also determine the driving force for nucleation (Δμ_N_^EC^) at 185 MPa as a function of the supercooling degree and the H_2_ hydrate occupancy. We determine that, thermodynamically, the most favored occupancy of the H_2_ hydrate consists of 1 H_2_ molecule in the D cages and 3 in the H cages (i.e., 1–3 occupancy). We also conclude that the double occupancy of the small D cages is not favored because the Δμ_N_^EC^ values obtained for this occupancy are the most positive ones. The second approach used in this work is the direct coexistence technique using an initial H_2_ hydrate phase with 1–3 occupancy. We also propose a new modification of the Berthelot combining rule to improve the predictions of the *T*_3_ values. Following this method, we determine the *T*_3_ at 100, 185, and 300 MPa finding excellent agreement with the experimental data.

## Graphical abstract

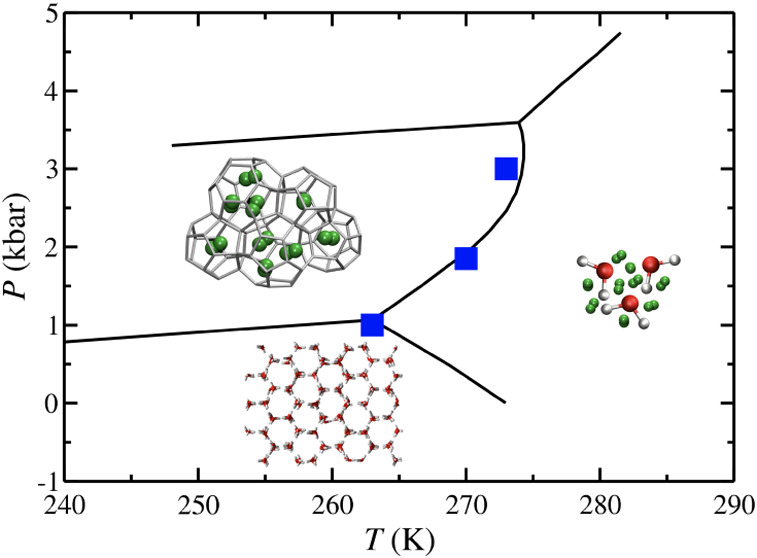

## Introduction

Clathrates are nonstoichiometric inclusion crystalline compounds consisting of a network of hydrogen-bonded molecules (host) forming cages in which small molecules (guest) such as hydrogen (H_2_), nitrogen (N_2_), carbon dioxide (CO_2_), and methane (CH_4_), among many others, can be encapsulated under the appropriate thermodynamic conditions.^1,2^ When water acts as the host molecule, these structures are specifically termed clathrate hydrates or simply hydrates. Among the many hydrates applications such as CO_2_ capture^3–10^ or N_2_ recovery from industrial emissions,^11,12^ it is interesting to remark on their energetic applications. In nature, vast deposits of natural gas exist as hydrates. According to the last estimations, conventional natural gas deposits correspond to 20% of the total natural gas present in nature, while the remaining 80% is trapped as hydrate on the seabeds and permafrost areas.^13–15^ Thus, methane hydrate represents an interesting alternative as a source of energy.

Although the use of hydrates as a source of natural gas would help to alleviate the global energy crisis that the world is facing today, it is also necessary to mitigate anthropogenic carbon emissions in order to palliate climate change. However, reducing carbon emissions while ensuring the energy needs of a growing population are met is not an easy task. In this regard, the use of hydrates of H_2_, in combination with hydrate promoters, as strategic materials for gas transport and storage, is one of the most significant and promising applications of hydrates in the environmental, energetic, and economic context. This represents a potential alternative to metal hydrides that are currently in use. However, the use of metal hydrides as H_2_ storage media has not yet been implemented due to a lack of information regarding the thermodynamic and kinetic properties of these compounds.^11,16–18^ The utilization of H_2_ hydrates in this context would result in a reduction in raw material costs while maintaining a comparable volumetric storage capacity. In order to achieve this, it is necessary a deep understanding of the phase equilibria and the kinetics of the formation and growth of these hydrates, with special emphasis on which are the factors that rule the occupancy of these hydrates.^17–34^

At high pressures and low temperatures, H_2_ hydrates crystallize in the so-called sII structure with 136 water molecules distributed in 16 D (pentagonal dodecahedron or 5^12^) cages and 8 H (hexakaidecahedron or 5^12^6^4^) cages. The D, or small, cages are better stabilized by small molecules such as H_2_ or N_2_ while the H, or large, cages are better stabilized by larger molecules such as propane. However, it is interesting to remark that the large H cages can be also stabilized by the multiple occupancy of small molecules such as H_2_ or N_2_.^1,2^ From an experimental point of view, the analysis of hydrate occupancy is a challenging endeavor, primarily due to the extreme conditions required for their formation and equilibrium stability. These conditions include high pressure and low temperature, which present significant difficulties in producing homogeneous samples for analysis. Furthermore, the experimental measurement of equilibrium properties, such as the lattice constants and cage occupancies, is not a straightforward process.^19,21–24,35^ In a series of recent works, Zhang et al.^36–38^ provide a detailed analysis of the H_2_ hydrate formation in the presence of several hydrate promoters (tetrahydrofuran, l-val, and 1,3-dioxolane). They studied the formation kinetics of the H_2_ hydrate under the presence of different thermodynamic and kinetic promoters as well as a detailed analysis of how the hydrate promoter concentration affects the H_2_ hydrate formation. They study the occupancy of both types of sII hydrate cages by using Raman spectroscopy, which is a special interesting topic due to the possibility of using hydrates to store H_2_, concluding that H_2_ molecules solely occupy the small D cages of the sII hydrates with thermodynamic hydrate promoters occupying the large H cages. At this point, it is interesting to remark that although tetrahydrofuran is a very special thermodynamic hydrate promoter since it is able to form a hydrate by itself,^13,39–49^ the 1,3-dioxolane is less toxic^50^ and, hence, more friendly from an environmental point of view, which is a prerequisite for large-scale application of hydrate-based H_2_ storage technology.^38^

In order to use hydrates as H_2_ storage media, it is necessary to obtain a deep understanding of how the thermodynamic conditions affect the occupancy of these compounds since an increment of the occupancy implies an improvement of the storage capacity. In this respect, theoretical and simulation approaches provide an interesting molecular perspective to study the occupancy of these hydrates. Although the occupancy of H_2_ hydrates has been the subject of several studies, there is still an open debate about the amount of H_2_ that can be encapsulated inside the small, D, and large, H cages of the sII hydrate structure. From an experimental point of view, Mao et al.^19,24^ state that the double occupancy of both types of cage is possible. This has been corroborated by Belosudov et al.^20^ from theoretical calculations and by Liu et al.^27^ from ab initio computations. However, from simulations, there is still controversy over the H_2_ hydrate occupancy. Alavi et al.^29^ state that, at low pressures (below 2.5 kbar), the most stable occupancy of the sII H_2_ hydrate consists of 4 H_2_ molecules in the H cages while the D cages remain single occupied. They also remark that the double occupancy of the D cages provokes an increase in the structure energy and tetragonal distortions of the hydrate unit cell. The same conclusion was found by Papadimitriou et al.^30^ at pressures between 380 and 450 MPa. They also affirm that the most stable occupancy consists of 4 and 1 H_2_ molecules in the H and D cages, respectively. They also study the effect of lattice constant on the storage capacity of hydrogen hydrates^26^ and the impact of different force fields on the H_2_ hydrate storage predictions.^33^ Also in the works of Katsumasa et al.^25^ and Chun and Lee^32^ it is shown that H cages can be multiply occupied while D cages remain single occupied in most cases. Contrary to the previous simulation studies, Brumby et al.^17^ performed a detailed analysis of the H_2_ hydrate occupancy from Gibbs ensemble Monte Carlo simulations. They concluded that the occupancy of D cages is not limited to only single occupancy at pressures below 400 MPa, although only a small percentage of them in the hydrate structure were doubly occupied.

In this work, we study the dissociation temperature of the sII H_2_ hydrate at 185 MPa as a function of the multiple occupancy of both the D and H cages. At the dissociation temperature (*T*_3_), the system under study presents a three-phase equilibrium. The three phases involved are a H_2_ hydrate phase, an aqueous phase with the corresponding equilibrium H_2_ solubility, and a pure H_2_ phase. The dissociation temperature at 185 MPa and for various H_2_ hydrate occupancies are obtained using molecular dynamics simulations and the solubility method.^51–55^ We use the TIP4P/Ice^56^ and a modified version of the Silvera and Goldman^29,57^ models to describe the molecules of water and H_2_ respectively. We analyze the effect of the occupancy of the large H cages from single to quadruple occupancy and the occupancy of the small D cages from single to double occupancy on the dissociation temperature. We also obtained the driving force for nucleation, Δμ_N_, at 185 MPa as a function of the H_2_ hydrate occupancy and the supercooling degree. Taking into account the dissociation temperature and the driving force for nucleation results obtained for the different occupancies, we study the dissociation temperature of the most favored one (3 and 1 H_2_ molecules in the H and D cages, respectively) from 100 to 300 MPa. Finally, we propose a new modification of the Berthelot combining rule to improve the simulation results and predict accurately the experimental data.^21,35^ It is important to remark that, experimentally, hydrate phase diagrams are studied by performing experiments at different thermodynamic conditions and monitoring when a phase transition takes place.^21,35^ Notice that this implies the crystallization of the H_2_ hydrate from an H_2_ aqueous solution. Homogeneous nucleation at the thermodynamic equilibrium conditions is a rare event that can only be studied from simulation using special and computationally expensive techniques, which hinders employing the same specific conditions and procedures as experiments. However, the same information can be obtained by simpler and cheaper simulation methodologies such as the direct coexistence technique^11,18,48,58–66^ and the solubility method.^51–55^ Notice that, for a given pressure, there is only a possible *T*_3_ value that should be independent of the specific conditions at which experiments and simulations are performed.

The organization of this article is as follows: In the Models, Simulations Details, and Methodology section, we describe the models, simulation details, and methodology used in this work. The results obtained, as well as their discussion, are described in the Results section. Finally, the conclusions are presented in the Conclusions section.

## Models, Simulation Details, and Methodology

In this work, all molecular dynamics simulations have been carried out using the GROMACS[Bibr ref67] (2016.5 double–precision version) software package. Water molecules are modeled using the widely known TIP4P/Ice model.^56^ Following the work of Alavi et al.,^29^ we employ a modified version of the Silvera and Goldman^57^ (SG) H_2_ model based on the well depth and potential minimum of the original SG isotropic isolated pair potential for gas-phase hydrogen. This modified version simplifies the original SG interaction potential and makes it more suitable for high-performance simulations.^18^ In this model, the H_2_ molecule is described by two hydrogen atoms linked by a rigid bond of 0.7414 Å with a Lennard–Jones (LJ) interactive site in the molecule’s center of mass. Also, positive charges are placed on the hydrogen centers while the negative charge is placed in the molecule’s center of mass together with the LJ interactive site. A summary of the molecular model details of both compounds is shown in Table 1.

**Table 1:** Nonbonded Interaction Parameters and Geometry Details of TIP4P/Ice Water^56^ and Modified SG H_2_ ^18,29,68^ Molecular Models Employed in This Work

atom	σ (Å)	ε/*k*_B_ (K)	*q* (e)	geometry details	
Water (TIP4P/Ice)					
O	3.1668	106.1	-	*d*_OH_ (Å)	0.9572
H	-	-	0.5897	H–O–H (deg)	104.5
M	-	-	–1.1794	*d*_OM_ (Å)	0.1577
H_2_ (SG)					
H	0	0	0.4932	*d*_HH_ (Å)	0.7414
mass center	3.038	34.302	–0.9864		

In all cases the nonbonded cross interactions between unlike groups are calculated using the Lorentz–Berthelot combining rule. However, as in the original work of Michalis et al.,^18^ we modify the Berthelot combination rule between the oxygen group from the water molecule and the LJ interactive site from the H_2_ molecule. This modification improves the predictions of the H_2_ solubility in water and the three-phase coexistence temperature (*T*_3_) determination in the 273.15–323.15 K and 100–300 MPa range of temperatures and pressures, respectively. In particular, the Berthelot combination rule is modified by a χ factor whose value is a function of the temperature

1 $$\epsilon_{\mathrm{O-}{\mathrm{MH}}_{2}}=\chi (\epsilon_{\mathrm{OO}}\epsilon_{{\mathrm{MH}}_{2}{\mathrm{-MH}}_{2}}{)}^{1/2}$$

2 $$\chi =a_{1}\exp\left(-\frac{T}{a_{2}}\right)+a_{0}+b_{0}$$

where χ is the Berthelot modifier factor, ϵ_OO_ and ϵ_MH_2_–MH_2__ are the LJ well-depth associated with the oxygen atom from the water molecule and the center of mass of the H_2_ molecule, respectively. The *a*_0_, *a*_1_, and *a*_2_ parameters are taken from the work of Michalis et al.,^18^ and the *b*_0_ parameter value is proposed in this work. Particularly, the model proposed by these authors corresponds to values *a*_0_ = 0.649194, *a*_1_ = 0.12085, and *a*_2_ = −186.29458 K. Notice that when *b*_0_ = 0, eq 2 is identical to that proposed by Michalis et al.^18^

Although the original expression for the χ factor proposed by Michalis et al.^18^ provides an excellent agreement between the experimental and the simulated solubility of H_2_ in water, and also improves the prediction of the three-phase coexistence temperature (*T*_3_) in the 100–300 MPa range of pressure, the *T*_3_ values obtained from simulation still underestimate the experimental *T*_3_ value (see Direct Coexistence Method section for further details). Hence, in this work, we propose a new χ expression based on the original one proposed by Michalis et al.^18^ We introduce the *b*_0_ parameter in eq 2 in order to improve the *T*_3_ value obtained from molecular dynamic simulations. In this work, *b*_0_ has a value of 0.1. The addition of this factor provides an excellent prediction of the H_2_ hydrate *T*_3_ value but slightly overestimates the H_2_ solubility in water. This *b*_0_ value was obtained by determining the predicted *T*_3_ value by using different χ values. In all cases, we used the initial value obtained from the original χ expression, and *T*_3_ was evaluated at 100, 185, and 300 MPa by systematically varying χ in increments of 0.1. We determine that just a 0.1 increment is enough to provide an accurate description of the *T*_3_ value since an additional increment of the χ factor (*b*_0_ = 0.2) overestimates it. A detailed analysis of the solubility of H_2_ in water using the TIP4P/Ice and SG models for the water and H_2_ molecules as a function of the χ factor can be found in the original work of Michalis et al.^18^

In this work, the Verlet leapfrog^69^ algorithm with a time step of 2 fs is used to solve the motion equations of Newton. Also, we use the Nosé–Hoover thermostat,^70^ with a time constant of 2 ps, and the Parrinello–Rahman barostat,^71^ with a time constant of 2 ps and a 5 × 10^–5^ compressibility value, to ensure that simulations are performed at constant temperate and pressure. Following the original work of Michalis et al.,^18^ we use a cutoff value of 1.1 nm for the Coulombic and dispersive interactions. We do not use long-range corrections for the dispersive LJ interactions but Particle-Mesh Ewald (PME)^72^ corrections are used for the Coulombic potential.

In order to determine the three-phase coexistence temperature, we employed two different methods. First, we employ the solubility method^51–55^ and the original modification of the χ factor proposed by Michalis et al.^18^ (i.e., *b*_0_ = 0). Following the procedure used in our previous works,^52–55^ the *T*_3_ value is determined by studying the solubility of H_2_ in an aqueous solution phase when it is in contact via a planar interface with an initial pure H_2_ phase (L_w_–L_H_2__ two-phase equilibria) and when in contact with a hydrate phase (H–L_w_ two-phase equilibria). The number of molecules used in each case is specified in the corresponding sections. According to the solubility method, the *T*_3_ value is determined by representing the solubility values obtained from both equilibria at a constant pressure and as a function of the temperature. The solubility of H_2_ in the aqueous phase, when it is in contact with the H_2_-rich liquid phase, decreases with temperature, as it happens with the solubility of most gases in water; in contrast, the solubility of H_2_, when it is in contact with the hydrate phase, increases with temperature. Note that this behavior with temperature is similar to that observed when a solid is dissolved in water. In the intersection of both curves, the aqueous phase has reached the same thermodynamic equilibrium state (monitoring by the H_2_ solubility value) when in contact with a H_2_ hydrate phase and when in contact with a pure liquid H_2_ phase separately, i.e., the temperature at which both curves cross is the temperature at which the liquid water, hydrate, and H_2_ liquid phases are in equilibrium, which represents the so-called *T*_3_ at the corresponding pressure. In this work, the *T*_3_ value is determined using the solubility method and the χ factor proposed by Michalis et al.^18^ at *P* = 185 MPa. We also analyze the effect of the H_2_ hydrate occupancy on the *T*_3_ value. The sII unit cell hydrate structure is built up by 16 D (small) and 8 H (large) hydrate cages. In this work, we calculated the *T*_3_ at 185 MPa when the D, or small, cages are singly occupied and the H, or large, cages are occupied by 1, 2, 3, and 4 H_2_ molecules (called from now on 1–1, 1–2, 1–3, and 1–4 occupancies). Also, we analyze the effect of double occupancy in the small D cages when 2 and 4 H_2_ molecules occupy the small D and large H cages respectively (2–2 and 2–4 occupancies). Unfortunately, although we generate and equilibrate a bulk 2–4 H_2_ hydrate phase, it becomes unstable when it is put in contact with an aqueous phase. Therefore, we conclude that the 2–4 occupancy is not stable and can not be simulated under the thermodynamic conditions considered in this work.

The L_w_–L_H_2__ equilibria simulations are performed in the *NP*_*z*_ *T* ensemble, i.e., only the *P*_*z*_ component of the pressure tensor, which is perpendicular to the L_w_–L_H_2__ planar interface, is fixed by the barostat. The H–L_w_ equilibria simulations are carried out in the anisotropic *NPT* ensemble i.e., each side of the simulation box is allowed to fluctuate independently to keep the pressure constant and to avoid any stress from the solid hydrate structure. Also, we perform extra bulk simulations, in the isotropic *NPT* ensemble, of pure water, pure H_2_, and pure H_2_ hydrate phases with different occupancy levels to determine the driving force for nucleation as a function of the H_2_ hydrate occupancy and the supercooling degree. The water and H_2_ pure bulk phases are built up by 1000 molecules of water and H_2_, respectively, and the bulk H_2_ hydrate phase is obtained by replicating the unit cell twice in each space direction taking into account the corresponding H_2_ occupancy.

The second method used in this work is the direct coexistence technique.^11,18,48,58–65,73–80^ Following this method, the three phases involved in the equilibrium (H–L_w_–L_H_2__) are placed together in the same simulation box. By fixing the pressure and varying the temperature, one can analyze the behavior of the three-phase system and determine the *T*_3_ value. If the fixed temperature is below the *T*_3_, at a given pressure value, the aqueous L_w_ phase becomes unstable and the hydrate phase grows until extinguishing the L_w_ or the L_H_2__ phase depending on the number of molecules of water and guest in both phases. Contrarily, if the fixed temperature is above the *T*_3_ value, the hydrate phase becomes unstable and it melts, obtaining a L_w_–L_H_2__ equilibria. In this work, the *T*_3_ value is obtained at three different pressures (100, 185, and 300 MPa), using an initial hydrate phase seed with a 1–3 occupancy and using the modified χ factor expression proposed in this work (eq 2 with *b*_0_ = 0.1). As in the study of the H–L_w_ equilibria, simulations are performed in the anisotropic *NPT* ensemble to avoid any stress from the solid hydrate structure.

## Results

In this section, we present a detailed analysis of the H_2_ hydrate dissociation temperature. First, we focus on determining the *T*_3_ value at 185 MPa as a function of the H_2_ hydrate occupancy using the solubility method and a modification of the Berthelot combining rule already presented in the literature by Michalis et al.^18^ We also study the driving force for nucleation of the H_2_ hydrate as a function of the supercooling degree for the five different occupancies studied in this work at 185 MPa. Finally, we propose a modification of the χ factor expression proposed by Michalis et al.^18^ and we determine the *T*_3_ at 100, 185, and 300 MPa using the direct coexistence technique. The new expression for the χ factor proposed in this work provides the same *T*_3_ values from simulations as those reported from experiments in the literature between the error bars. At this point, it is important to remark that the χ original factor expression was proposed to match the simulated and experimental solubility of H_2_ in an aqueous phase. This resulted in an improvement of the *T*_3_ prediction using the classical Lorentz–Berthelot combining rule, but the *T*_3_ values obtained with this modification of the Berthelot combining rule still slightly underestimate the experimental *T*_3_ value. On the other hand, the new expression for the χ factor proposed in this work provides an excellent prediction of the *T*_3_ values but slightly overestimates the H_2_ solubility in water.

### Solubility Method

In order to determine the *T*_3_ value at 185 MPa through the solubility method, first it is necessary to obtain the solubility of H_2_ in water when an aqueous phase (L_w_) is in contact with a pure H_2_ phase (L_H_2__) and when in contact with a H_2_ hydrate phase (H). First, we focus on the L_w_–L_H_2__ equilibria and then in the H–L_w_ equilibria.

#### L_w_–L_H_2__ Equilibria: Solubility of H_2_ in Liquid Water and Interfacial Tension

We study the L_w_–L_H_2__ equilibria behavior by running *NP*_*z*_ *T* simulations at 185 MPa and 230, 240, 250, 260, 270, and 280 K. The initial simulation box is built up by a pure water phase with 2800 water molecules and a pure H_2_ phase with 1400 H_2_ molecules. Both phases are put in contact via a planar interface along the *z*-direction. The *xy* interfacial area remains constant throughout the whole simulation since the values of *L* = *L_x_* = *L_y_* are fixed at 3.8 nm. Finally, the average *L*_*z*_ side of the simulation box is ≈9.8 nm and both phases approximately fill half of the simulation box along the *z*-axis direction. Due to the low solubility of H_2_ in water, simulations are run for 800 ns. The first 400 ns are taken as the equilibration period, and the last 400 ns are taken as the production period.

The H_2_ solubility values in the aqueous phase are calculated from the analysis of the density profiles. These are obtained by dividing the simulation box into 200 slabs and assigning the center of mass of each molecule to the corresponding slab. Finally, the mass density profile is obtained by multiplying the molar density profile of each compound by its corresponding molar mass. The solubility of H_2_ in the aqueous phase at each temperature is obtained by averaging for each component the values of the density profiles by using each slab belonging to the aqueous phase. The error bars of the densities of water and H_2_ in the aqueous phase at each temperature are obtained as the average standard deviation. The H_2_ solubility error bars are obtained by propagating the density errors obtained for both components. Calculations are performed far enough from the interface to avoid the effect of the interface on the average H_2_ solubility determination. Figure 1 shows the H_2_ solubility values in the aqueous phase obtained in this work at 185 MPa and several temperatures. The results obtained in this work are in good agreement with those reported by Michalis et al.^18^ As can be seen in Figure 1, the solubility of H_2_ in the aqueous phase decreases when the temperature is increased. This is the expected behavior of the solubility of a gas in water with the temperature. Notice that, when the temperature is increased, the H_2_ solubility varies more steeply at the lowest temperature values studied in this work.

**Figure 1:**
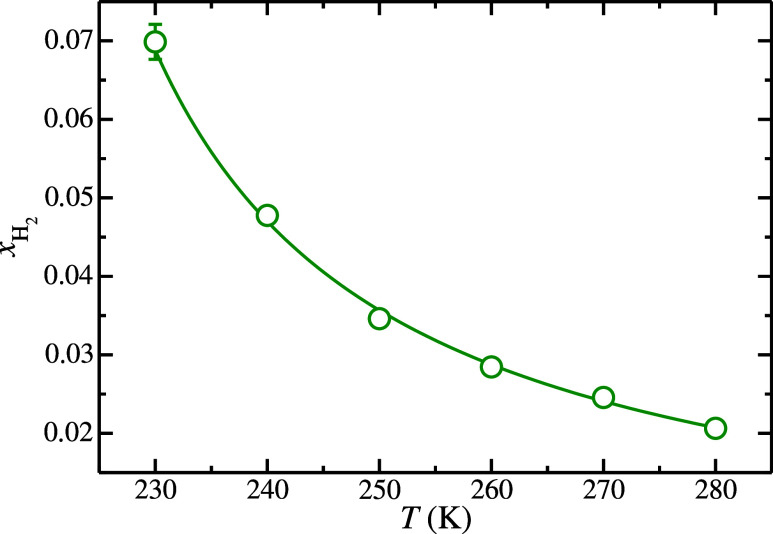
H_2_ solubility in the aqueous solution phase as a function of temperature at 185 MPa. The green open-circle symbols represent the solubility values obtained from the L_w_–L_H_2__ *NP*_*z*_ *T* molecular dynamic simulations performed in this work. The green curve is included as a guide for the eye.

From the analysis of the L_w_–L_H_2__ it is possible to determine the interfacial tension between the aqueous and the H_2_ phases from the diagonal components of the pressure tensor.^81–83^ As in the case of the density profiles, the interfacial tension at each temperature is determined from the last 400 ns of the L_w_–L_H_2__ molecular dynamic simulations. In order to obtain an estimation of the errors, the 400 ns of the production period are divided into 10 blocks of 40 ns. The final value of the interfacial tension is obtained by averaging the value of each block. Finally, uncertainties are estimated as the standard deviation of the average.^84^ As can be observed in Figure 2, the L_w_–L_H_2__ interfacial tension decreases steeply when the temperature is increased from 230 to 260 K until reaches an almost plateau value at 270 and 280 K. Similar behavior was found in a previous work^54^ when the liquid water–liquid nitrogen (L_w_–L_N_2__) interfacial tension was analyzed at 1000 and 1500 bar from 245 to 300 K. Unfortunately, as far as the authors know, there are no experimental data available in the literature to compare with the results obtained in this work.

**Figure 2:**
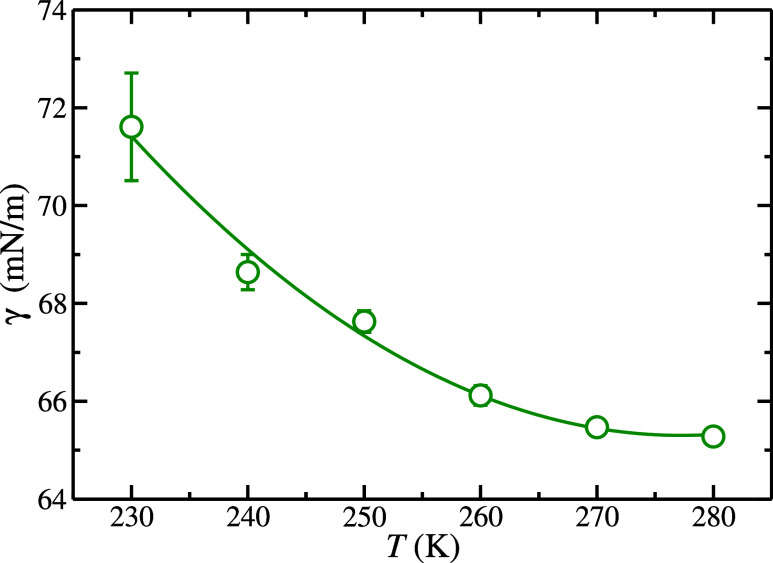
L_w_–L_H_2__ interfacial tension, γ, as a function of the temperature at 185 MPa. The results obtained in this work from molecular dynamic *NP*_*z*_ *T* simulations, and its error bars, are represented as open green circles. The green curve is included as a guide for the eyes.

#### H–L_w_ Equilibria: Solubility of H_2_ in Liquid Water When It Is in Contact with H_2_ Hydrate

Following an approach similar to that in the L_w_–L_H_2__ equilibria, we study the solubility of H_2_ in an aqueous phase when it is in contact via a planar interface with a H_2_ hydrate phase (H–L_w_). We obtain the H_2_ solubility values at 185 MPa from 250 to 270 K by running *NPT* simulations. The barostat is applied anisotropically in the three space directions to avoid stress from the solid H_2_ hydrate structure. Simulations are run for 800 ns, with the first 200 ns being considered as the equilibration period and the last 600 ns being considered as the production period. In this case, the initial simulation box is prepared as follows. The hydrate is formed from 1088 molecules of water and a different number of H_2_ molecules depending on the occupation level (see Table 2). As can be seen, this corresponds to a 2 × 2 × 2 unit cell of the sII hydrate structure with different occupancies and space groups of the unit cell *Fd*3*m*. The proton disorder is obtained using the algorithm of Buch et al.^85^ Once the hydrate phase is equilibrated, it is put in contact with a water-liquid phase.

**Table 2:** Initial Number of Molecules of Water and H_2_ in Each H–L_w_ Simulation Box at Each H_2_ Hydrate Occupancy and Temperature^a^

		hydrate phase			
H_2_ hydrate occupancy	*T* (K)	unit cell	water	H_2_	L_w_ phase water
1–1	250	2 × 2 × 2	1088	192	2176
	260				
	265				
1–2	250	2 × 2 × 2	1088	256	2176
	260				
	265				
	270				
2–2	250	2 × 2 × 2	1088	384	6528
	260				6528
	265				6528
	270				4352
1–3	250	2 × 2 × 2	1088	320	4352
	260				4352
	265				4352
1–4	250	2 × 2 × 2	1088	384	6528
	260				6528
	265				6528
	270				4352

a In all cases, the initial L_w_ phase contains only water molecules.

Due to the low solubility of H_2_ in water, the initial aqueous phase in contact with the H_2_ hydrate phase is built up as an initial pure water phase. Both phases are in contact via a planar interface along the *z*–direction. In order to reach the equilibrium solubility of H_2_, part of the initial hydrate phase has to melt and release H_2_ into the aqueous phase. This procedure is far from being arbitrary since it ensures that the hydrate phase is not going to grow along the simulation, and hence, the hydrate phase that remains in the simulation box, once the system has reached equilibrium, has the same occupancy (stoichiometry) as the initial one. This procedure has been recently used by some of the authors of this work to study the three-phase dissociation temperature of the N_2_ hydrate when it presents single (1–1) and double (1–2) occupancies.^55^

From the analysis of the density profiles, it is possible to determine not only the H_2_ solubility in the aqueous phase but also whether the hydrate phase melts or grows. As has been remarked previously, the initial aqueous phase only contains water molecules, and the only way to reach the equilibrium H_2_ solubility is that part of the H_2_ hydrate melts. Technically, the H_2_ hydrate phase can not grow since there is no H_2_ in the initial aqueous phase and an empty hydrate phase is not stable. However, when the occupancy of H_2_ molecules in the hydrate is high enough and part of the hydrate melts, it could release into the aqueous phase more H_2_ than necessary to reach the solubility of equilibrium, oversaturating the aqueous phase with H_2_. This happens as a consequence of the low solubility of H_2_ in the aqueous phase and the high amount of H_2_ stored in the hydrate structure. When this happens, the hydrate phase grows to take back the excess H_2_ from the aqueous phase. If the hydrate phase grows, we can not ensure that the stoichiometry of the new hydrate phase is the same as the initial one and, hence, we can not ensure that the solubility of H_2_ in the aqueous phase is reached when the aqueous phase is in contact with a H_2_ hydrate phase at only the desire H_2_ hydrate occupancy. To avoid oversaturation of the aqueous phase when the H_2_ hydrate melts, the number of molecules of water in the initial aqueous phase increases when the occupancy of the H_2_ hydrate is increased (see Table 2 for further details). In order to ensure that the stoichiometric of the hydrate along the simulation is the same as the initial seed, we carefully monitor the hydrate phase evolution by the analysis of the density profiles along the simulation. Density profiles provide an accurate description of the system distribution. The peaked shape region of the hydrate density phase contrasts with the flat density region of the fluid phase. By monitoring the length of the hydrate phase, it is possible to determine if the hydrate phase grows, melts, or both at different simulation times. In all cases, the hydrate phase only melts, ensuring that the remaining hydrate phase has the same stoichiometric as the initial seed. At this point, it is also important to mention that there is no diffusion of the guest through the hydrate phase and, as a consequence of this, the only option to modify the initial stoichiometric of the hydrate is the growing of this one with a different occupancy level. Also, it is important to take into account that the H_2_ solubility in the aqueous phase in contact via a planar interface with the H_2_ hydrate phase increases with the temperature. It means that when the temperature increases, and if the initial aqueous phase is big enough, the H_2_ hydrate phase could melt completely in order to reach the equilibrium H_2_ solubility value. For this reason, the number of water molecules in the initial aqueous phase is not the same at different temperatures, even when the occupancy of the H_2_ hydrate phase is the same (see, for example, the simulation boxes used for 2–2 and 1–4 occupancies in Table 2). Obviously, an increase in the number of molecules involves an increase in the computational effort required to perform the simulations. Hence, the number of water molecules in the aqueous phase is increased only if it is strictly necessary to ensure the stoichiometry of the H_2_ hydrate phase.

The H_2_ solubility in the aqueous phase is obtained following the same procedure as that in the case of the L_w_–L_H_2__ equilibria. First, the density profiles of water and H_2_ are obtained from each initial H_2_ hydrate occupancy and temperature by dividing the simulation box into 200 slabs along the direction perpendicular to the interface (the *z*-axis). The center of mass of each molecule is assigned to its corresponding slab, and the mass density profile is obtained by multiplying the molar density profile of each component by its corresponding molar mass. As it has been explained previously, the H_2_ solubility in the aqueous phase at each H_2_ hydrate occupancy and temperature is obtained by averaging the water and H_2_ density profile values of each slab belonging to the aqueous phase. The error bars of the water and H_2_ densities in the aqueous phase at each temperature are obtained as the average standard deviation. The H_2_ solubility error bars are obtained by propagating the density errors obtained for both components. Again, calculations are performed far enough from the H–L_w_ interface to avoid any unwanted interfacial effect on the aqueous bulk solubility of H_2_. The results obtained in this work for each H_2_ hydrate occupancy are presented in Figure 3. As we can see, the solubility of H_2_ in the aqueous phase when in contact with a H_2_ hydrate phase via a planar interface increases when the temperature is increased. This is the expected behavior when the temperature increases since the hydrate phase becomes less stable and part of it melts, releasing more H_2_ into the aqueous phase. The solubility of H_2_ in the aqueous phase, when it is in contact with a hydrate phase, can be understood as a special case of the solubility of a solid in water. Typically, the solubility of a solid in water increases when the temperature increases. In the case of hydrates, the same behavior is expected. However, hydrates are a special case since when part of their structure melts into the aqueous phase, two components are released: water and the guest. Since the solvent is water, the molecules of water from the hydrate become part of the proper solvent of the aqueous solution, while the guest molecules become the solute. As we explained previously, the initial L_w_ phase is basically a pure water phase, which means that all the H_2_ present in this phase comes from the H_2_ hydrate phase. It is interesting to remark that the solubility of H_2_ in the aqueous phase seems to be almost independent of the H_2_ hydrate occupancy since all the systems studied in this work present similar H_2_ solubility values. It is also interesting to point out that, in the range of temperatures studied in this work, the H_2_ solubility increases linearly when the temperature is increased.

**Figure 3:**
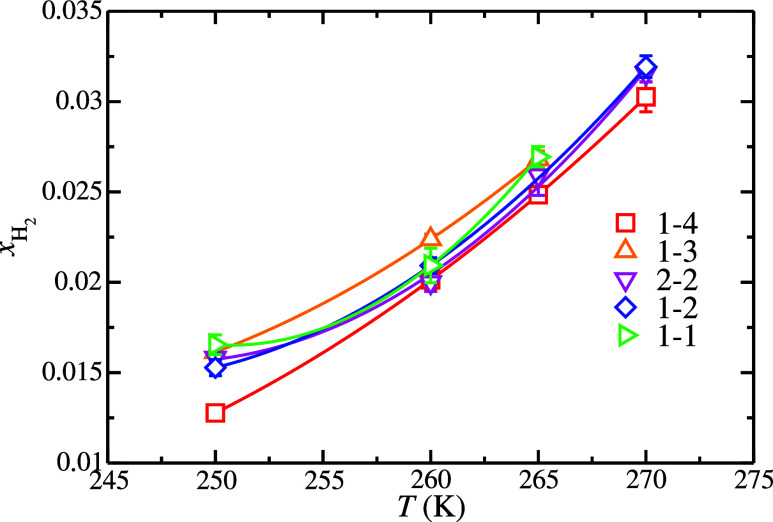
H_2_ solubility in the aqueous solution phase as a function of temperature at 185 MPa. The symbols represent the solubility values obtained from the H–L_w_ *NPT* molecular dynamic simulations performed in this work at different H_2_ hydrate occupancies. The meaning of the symbols is given in the legend. Each curve is obtained from the fitting of the solubility value at each occupancy and is included as a guide for the eyes.

#### Three-Phase Coexistence Line (*T*_3_) Determination from the Solubility Method

From the analysis of the solubility of H_2_ in the aqueous phase obtained from the L_w_–L_H_2__ and H–L_w_ equilibria, it is possible to determine the temperature (*T*_3_) at which the three phases (H–L_w_–L_H_2__) coexist in equilibrium. The *T*_3_ value is obtained as the temperature at which both H_2_ solubility curves cross at a certain pressure value. It is also possible to get an estimation of the *T*_3_ error bar by taking into account the error bars of the H_2_ solubility values obtained from both (L_w_–L_H_2__ and H–L_w_) equilibria. Figure 4 shows a representation of the method employed in this work to determine the error bars of the *T*_3_ values. For each equilibrium (L_w_–L_H_2__/H–L_w_), three H_2_ solubility curves are represented (red/blue). The central one represents the average H_2_ solubility value obtained at each temperature, while the upper/lower ones are obtained by summing/resting to the average H_2_ solubility value and its corresponding error. As a result, we obtain a central *T*_3_ value which corresponds to the temperature at which the central H_2_ solubility curves from both equilibria intersect, a lower *T*_3_^L^ value obtained as the intersection of the upper and lower H_2_ solubility curves obtained from the H–L_w_ and L_w_–L_H_2__ equilibria respectively, and an upper *T*_3_^U^ value obtained as the intersection of the lower and upper H_2_ solubility curves obtained from the H–L_w_ and L_w_–L_H_2__ equilibria respectively. Finally, the *T*_3_ error bar is obtained as (*T*_3_^U^ – *T*_3_^L^)/2. The results obtained for each occupancy are collected in Table 3 and the intersections of the solubility curves are presented in Figure 5.

**Figure 4:**
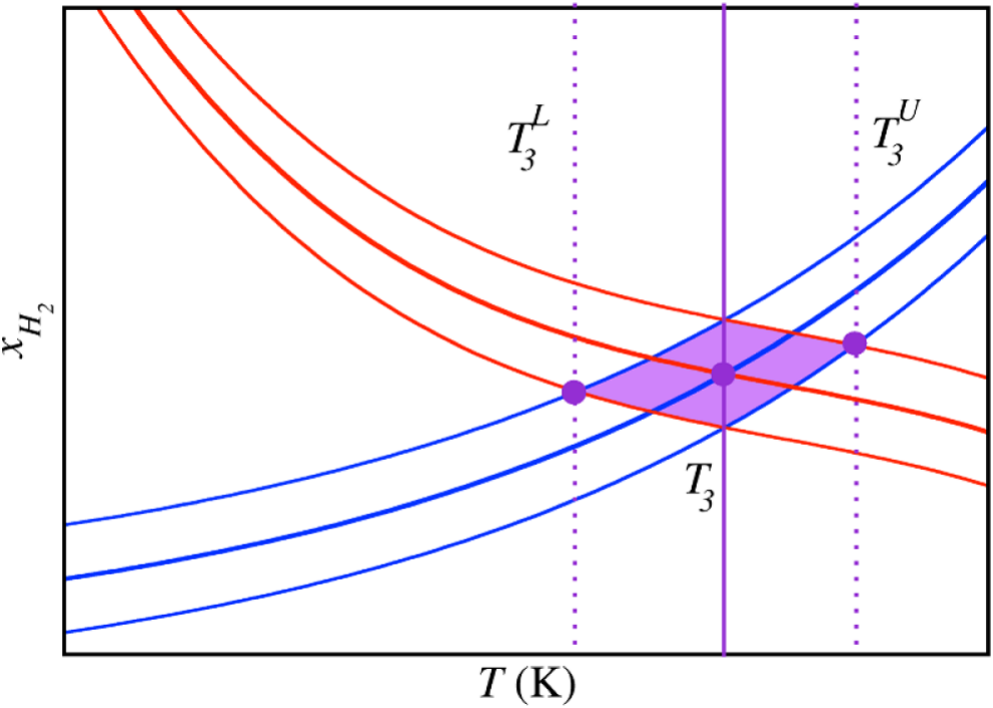
Schematic representation of the determination of the *T*_3_ value and its error bar. The three red and blue curves represent the solubility of H_2_ in the aqueous phase as a function of the temperature from the L_w_–L_H_2__ and H–L_w_ equilibria, respectively. In both cases, the central red/blue line represents the equilibrium H_2_ solubility, and the upper and lower red/blue lines represent the H_2_ solubility error bars. The violet-filled circles represent the lowest (*T*_3_^L^), average (*T*_3_), and upper (*T*_3_^U^) H_2_ solubility and temperature at which the H_2_ solubility curves cross.

**Figure 5:**
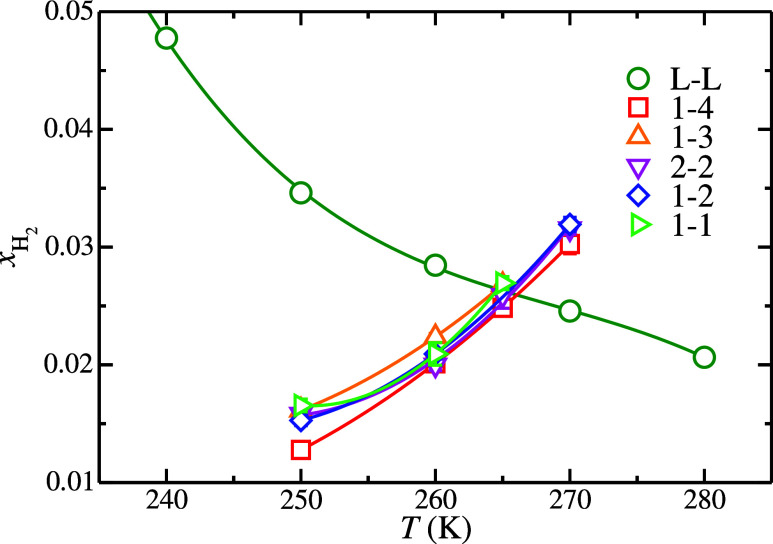
*T*_3_ values obtained at each H_2_ hydrate occupancy, as obtained from the solubility method. The meaning of the symbols is shown in the legend and it is the same as in the previous figures. The solubility curves are obtained by fitting the solubility H_2_ values in the aqueous phase and are included as a guide for the eyes.

**Table 3:** Dissociation Temperature, *T*_3_, of the H_2_ Hydrate, at Different H_2_ Hydrate Occupancies, as Obtained in This Work Using the Solubility Method at 185 MPa^a^

H_2_ hydrate occupancy	*T*_3_ (K)
1–1	264.6(6)
1–2	265.4(7)
2–2	265.6(8)
1–3	264.6(7)
1–4	266.1(8)

a Numbers in parentheses indicate the uncertainty of the results.

As shown in Figure 5 and Table 3, the H_2_ hydrate occupancy has an almost negligible effect on the final *T*_3_ value. All of the *T*_3_ values reported in this work at 185 MPa are the same, within the error bars, independently of the H_2_ hydrate occupancy. The *T*_3_ values obtained for each occupancy at 185 MPa are in agreement with that reported by Michalis et al.^18^ obtained using the TIP4P/Ice^86^ and Feynman–Hibbs (FH)^87^ models for water and H_2_ molecules, respectively. Although the H_2_ model (FH) employed in the original work of Michalis et al.^18^ to determine the H_2_ hydrate *T*_3_ value is different from the H_2_ model used in this work (SG),^57^ the same modifying factor of the Berthelot rule (eqs 1 and 2) for the water-H_2_ interactions has been employed in both works. This is so since Michalis et al.^18^ demonstrated that both H_2_ (FH and GS) models predict almost the same solubility of H_2_ in an aqueous phase, modeled by the TIP4P/Ice water model, when in contact with a pure H_2_ phase via a planar interface and the same modification of the Berthelot combining rule is required to match the experimental H_2_ solubility results. As well as in the work of Michalis et al.,^18^ the *T*_3_ values obtained in this study underestimate the experimental *T*_3_ values by 5–7 K.^21,35^ This is the expected result since it is similar to that obtained by Michalis et al.^18^ using the same modification of the Berthelot combining rule and the effect of the occupancy of the hydrate on the *T*_3_ value has been demonstrated in this work to be almost negligible.

As we will analyze in the next section, although all the H_2_ hydrate occupancies present the same *T*_3_ value, not all of them seem to be equally favored thermodynamically. Technically, it is necessary to calculate the free energy of each hydrate at the three-phase coexistence conditions to analyze which occupancy presents the lowest free-energy value and, hence, which one is the most stable. However, it is possible to estimate which occupancy is the most favored by analyzing the driving force for the nucleation behavior for each H_2_ hydrate occupancy. Although the driving force for nucleation represents a difference of free energies and not an absolute free-energy value, it gives us an approximated method to determine which H_2_ hydrate occupancy is the most favored under the thermodynamic conditions under which this study was carried out.

### Driving Force for Nucleation Δμ_N_^EC^

According to the Classical Nucleation Theory (CNT),^88^ homogeneous nucleation is an activated process, which means that in order to crystallize the system has to overcome a free-energy barrier. This free-energy barrier depends on the hydrate-aqueous solution’s interfacial free energy, γ, and the driving force for nucleation, Δμ_N_. The driving force for nucleation is defined as the difference between the chemical potential of water and H_2_ molecules in the hydrate phase and those in the aqueous solution phase. At the thermodynamic equilibrium conditions of temperature, pressure, and composition, the value of Δμ_N_ is 0 since the chemical potential of a molecule in each of the phases in equilibrium is the same. In the case of hydrates, the Δμ_N_ value becomes negative when the aqueous phase is supersaturated with the guest^52,89–93^ (H_2_ in this case) and when the temperature is below the dissociation temperature.^88,94,95^ When Δμ_N_ becomes more negative, the free-energy barrier that the system has to overcome to crystallize becomes smaller, i.e., it is possible to favor the homogeneous nucleation under supersaturation and/or supercooling conditions.

As we stated in our previous works,^53–55^ it is more convenient to express Δμ_N_ per cage of hydrate formed from the aqueous solution rather per guest molecule when the driving forces for nucleation of hydrates with different occupancies are compared. In previous works, some of the authors of this work presented a general expression for semi/multiple occupied hydrates.^53–55^ Following the same approach, the occupancy of the H_2_ hydrate is defined as *x*_occ_ = *n*_H_2__/*n*_cg_, where *n*_H_2__ and *n*_cg_ are the number of H_2_ molecules and cages per hydrate unit cell, respectively.^53,55^ According to Kashchiev and Firoozabadi,^94–96^ it is possible to describe the formation of a hydrate molecule from the aqueous solution phase as a chemical reaction at a certain value of *P* and *T*. This approach could be generalized to any H_2_ hydrate occupancy

3 $$x_{\mathrm{occ}}H_{2}\left(\mathrm{aq},x_{H_{2}}\right)+5.67H_{2}O\left(\mathrm{aq},x_{H_{2}}\right)\to {\left[{\left(H_{2}\right)}_{x_{\mathrm{occ}}}{\left(H_{2}O\right)}_{5.67}\right]}_{H}$$

H_2_(aq,*x*_H_2__) and H_2_O­(aq,*x*_H_2__) are the molecules of H_2_ and water in the aqueous solution phase with composition *x*_H_2__, respectively, while [(H_2_)_*x*_occ__(H_2_O)_5.67_]_H_ represents a “molecule” of the H_2_ hydrate in the solid phase with a certain occupancy, *x*_occ_. The 5.67 factor arises because the sII hydrate unit cell is formed by 136 molecules of water and 24 cages, and the “chemical reaction” of the H_2_ hydrate formation is reduced according to the number of hydrate cages. The same approach is used to calculate the molar enthalpy of a “hydrate molecule”, *h̃*_H_ = *H*/*N*_cg_, where *H* and *N*_cg_ are the total enthalpy of the H_2_ hydrate phase and the number of cages present in the hydrate structure, respectively. Although eq 3 is a general expression and can be applied to any thermodynamic condition, it is particularly interesting to apply this expression along the L_w_–L_H_2__ solubility curve, since most of the experiments on the nucleation of hydrates are performed when the water phase is in contact with the guest liquid phase through a planar interface. Following the notation of Grabowska *et al.*,^52^ the driving force for nucleation at experimental conditions is denoted as Δμ_N_^EC^. Also, it is important to take into account that under experimental conditions the *x*_H_2__ value of eq 3, at a given *P* value, is a function of the temperature. In this work, *x*_H_2__ has been obtained at 185 MPa and different temperatures (see Figure 1).

From eq 3 it is possible to define an approximated but simple method to calculate Δμ_N_^EC^.^52–55,94^ If eq 3 represents the formation of an H_2_ hydrate molecule, the inverse process corresponds to the dissociation of a H_2_ hydrate molecule into water and H_2_ molecules. Due to the low solubility of H_2_ in the aqueous phase, we can assume that the H_2_ hydrate molecules dissociate into pure water and H_2_. Taking this into account, the dissociation enthalpy of a hydrate molecule, *h̃*_H_^diss^, is defined as the enthalpy change of the hydrate dissociation in pure water and H_2_ molecules. In order to calculate Δμ_N_^EC^ in a simple way following the dissociation route approach,^52–55,94^ extra approximations have to be taken into account: (1) the solubility of H_2_ in the aqueous phase is assumed as 0 independently of the thermodynamic conditions, (2) *h̃*_H_^diss^ it is calculated at the *T*_3_ value, and (3) it is considered as a constant value independently of the temperature. Taking all these approximations, it is possible to define Δμ_N_^EC^ as

4 $$\Delta \mu_{N}^{\mathrm{EC}}\left(P,T,x_{\mathrm{occ}}\right)=k_{B}T\int_{T_{3}}^{T}\frac{{\mathrm{h̃}}_{H}^{\mathrm{diss}}\left(P,T^{\prime },x_{\mathrm{occ}}\right)}{k_{B}T^{\prime 2}}dT^{\prime }≊-{\mathrm{h̃}}_{H}^{\mathrm{diss}}\left(P,T_{3},x_{\mathrm{occ}}\right)\left(1-\frac{T}{T_{3}}\right)$$

Where *k*_B_ is the Boltzmann constant. Here it is important to remark that the occupancy of the hydrate is taken into account by the *h̃*_H_^diss^ value according to eq 3. Equation 4 has been named the dissociation route in our previous works.^52–55^

When the approximations assumed in eq 4 are not undertaken, the expression for the Δμ_N_^EC^ calculation becomes more complex since the solubility of H_2_ in the aqueous phase has to be explicitly taken into account as well as the change of *h̃*_H_^diss^ with *T* and *P*. As a result, we obtained the following expression named route 1 expression in our previous works^52–55^

5 $$\frac{\Delta \mu_{N}^{\mathrm{EC}}\left(P,T,x_{H_{2}}^{\mathrm{eq}},x_{\mathrm{occ}}\right)}{k_{B}T}=-\int_{T_{3}}^{T}\frac{{\mathrm{h̃}}_{H}^{H}\left(P,T^{\prime },x_{\mathrm{occ}}\right)-\left\{x_{\mathrm{occ}}h_{H_{2}}\left(P,T^{\prime }\right)+5.67h_{H_{2}O}\left(P,T^{\prime }\right)\right\}}{k_{B}T^{\prime 2}}dT^{\prime }-5.67\left[k_{B}T\ln\left\{x_{H_{2}O}^{\mathrm{eq}}\left(P,T\right)\right\}-k_{B}T_{3}\ln\left\{x_{H_{2}O}^{\mathrm{eq}}\left(P,T_{3}\right)\right\}\right]$$

Here *h̃*_H_^H^(*P*,*T*′,*x*_*occ*_) represents the enthalpy per cage of the hydrate at *P* and *T*′ with occupancy *x*_occ_ and *h*_H_2__ and *h*_H_2_O_ represent the molar enthalpy of pure H_2_ and water, respectively. We refer the reader to our previous works for further details.

Figure 6 shows the Δμ_N_^EC^ results obtained by both routes (eqs 5 and 4) as a function of the supercooling degree for the different H_2_ hydrate occupancies studied in this work. Although there are quantitative differences between the results obtained by both routes, the qualitative behavior of Δμ_N_^EC^ with the supercooling degree and the occupancy is the same. For the same H_2_ hydrate occupancy, Δμ_N_^EC^ becomes more negative when the supercooling degree is increased. This is the expected result, since the H_2_ hydrate phase becomes more stable than the aqueous phase as the temperature decreases. Also, for the same supercooling degree, Δμ_N_^EC^ becomes more negative when the amount of H_2_ in the H, or large, cages increases from 1 to 3 (from 1 to 1 to 1–3 occupancy), being the 1–3 occupancy of the most favored H_2_ hydrate occupancy, closely followed by the 1–2 occupancy. However, if the number of H_2_ molecules in the H cages is bigger than 3, then the H_2_ hydrate phase becomes slightly less favored and, as a consequence, Δμ_N_^EC^ increases becoming more positive. These results are in good agreement with those reported previously in the literature where the occupancy of the D and H cages of the H_2_ hydrate was studied from Monte Carlo^17,25,30,32^ and molecular dynamic^29^ simulations. In Figure 6 we can see that the Δμ_N_^EC^ results obtained, from both routes, for the 1–4 occupancy are between those obtained for the 1–1 and 1–2 occupancies. Also, we can state from Figure 6 that the double occupancy of the D, or small, cages destabilizes the hydrate structure due to the high repulsion present when two H_2_ molecules are confined in the D cages. As a consequence, the Δμ_N_^EC^ values obtained for the 2–2 occupancy are the most positive ones, revealing that among the different occupancies studied in this work, the 2–2 occupancy seems to be the less favored one. When considering hydrates as potential gas storage materials, the number of H_2_ molecules per cage is a crucial factor in determining the storage capacity of a given hydrate structure. Experimentally, it is known that the sII hydrate small cages could host two H_2_ molecules, while large cages could store up to four H_2_ molecules,^19^ leading to a total H_2_ storage capacity of 5.0 wt % for H_2_ hydrates. This H_2_ storage capacity is close to the US DOE 5.5 wt % target for 2025. However, our results indicate that the most stable hydrate configuration consists of three H_2_ molecules in the large cages and only one H_2_ molecule in the small one. This H_2_ hydrate stoichiometric results in a storage capacity of 3.2 wt %, which is roughly bigger than half of the US DOE ultimate target.

**Figure 6:**
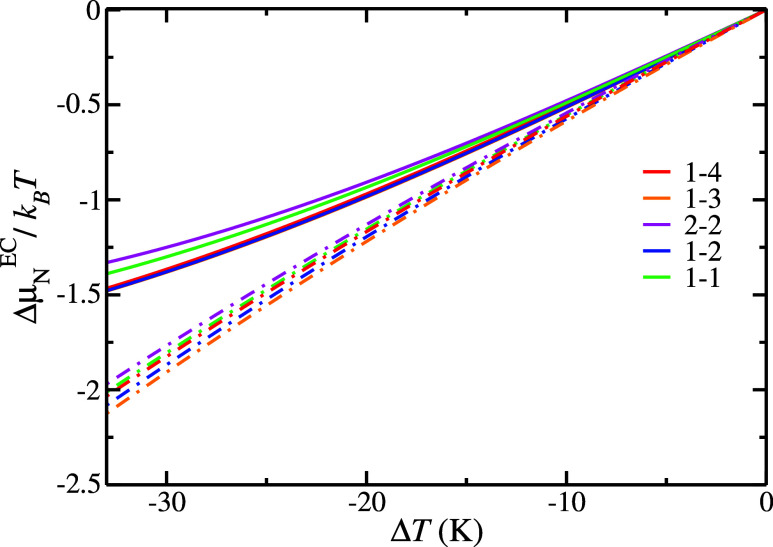
Δμ_N_^EC^ values for each H_2_ hydrate occupancy studied in this work as a function of the supercooling degree at 185 MPa. Continuous and dashed lines represent the results obtained by eqs 5 and 4 respectively.

### Effect of the Temperature and Occupancy on Unit-Cell Size

From the bulk simulations of the H_2_ hydrate phase used to determine the Δμ_N_^EC^, we analyze the average unit-cell size or lattice constant, *a* as a function of the temperature and the occupancy of the H_2_ hydrate phase at 185 MPa. Due to the cubic symmetry of the sII hydrate structure, we obtain *a* as the cubic root of the average volume of the hydrate unit cell. As shown in Figure 7, the H_2_ hydrate lattice constant increases when the temperature is increased, although the dependency of *a* with the temperature is almost negligible. This is an expected result since the same behavior was observed by some of the authors of this work for the case of the N_2_ hydrate.^55^ It is also interesting to analyze the behavior of *a* with H_2_ occupancy. As shown in Figure 7, *a* increases when the occupancy is increased. This is an expected behavior since a larger *a* value allows better accommodation of the H_2_ molecules inside the hydrate structure. As we can see, when the H_2_ occupancy increases from 1 H_2_ molecule in the H, or large, cages to 4 (i.e., from the 1–1 to 1–4 occupancies), the *a* value is increased by ≈0.7% in all the range of temperatures. It is also interesting to observe that when the D, or small, and the H, or large, cages are doubly occupied (2–2 occupancy), the *a* value is higher than in the 1–4 occupancy even when the total number of H_2_ molecules in the H_2_ hydrate unit cell is the same for both occupancies (48 H_2_ molecules). This is because the repulsion provoked by 2 H_2_ molecules encapsulated in the small D and large H cages is larger than the repulsion provoked by 4 H_2_ molecules in the large H cages and 1 H_2_ molecule in the small D cages.

**Figure 7:**
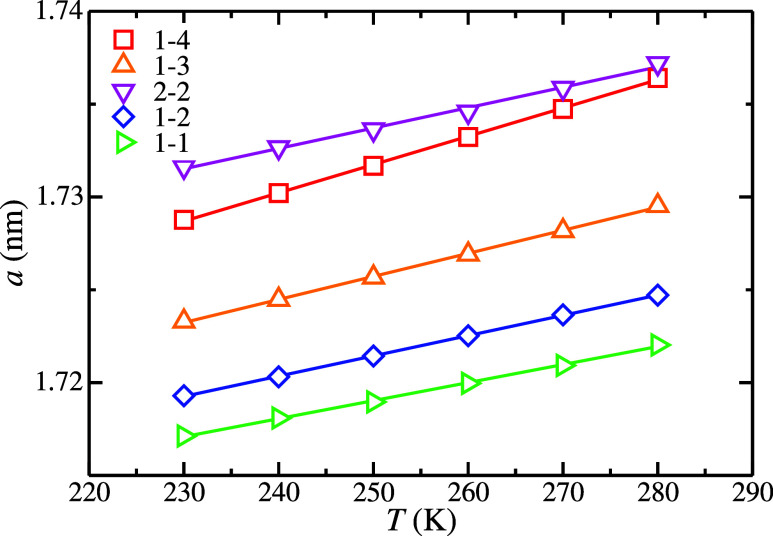
Average unit-cell size or lattice constant (*a*) obtained from MD *NPT* bulk simulations of the H_2_ hydrate phase as a function of *T* and the occupancy at 185 MPa. The meaning of the symbols is shown in the legend, and it is the same as in previous figures. The lines are included as a guide for the eyes.

### Direct Coexistence Method

Finally, we study the three-phase coexistence temperature, *T*_3_, using a direct coexistence method. Following this methodology, the three phases involved in the equilibrium are placed together in the same simulation box. As has been explained previously, at a given pressure value, two different behaviors are shown as a function of the temperature. If the temperature is above the *T*_3_ value, then the H_2_ hydrate phase is unstable and melts, evolving the three-phase system to a two-phase L_w_–L_H_2__ equilibrium. Contrarily, if the temperature is below the *T*_3_ value, the H_2_ hydrate phase grows until extinguishing the aqueous or guest phase depending on the initial amount of molecules of water and guest in both phases. To determine whether the H_2_ hydrate phase grows or melts, the potential energy of the system is monitored as a function of time. When the H_2_ hydrate phase grows, new hydrogen bonds are created, and the potential energy decreases. On the contrary, when the H_2_ hydrate phase melts, the potential energy increases as a function of time. The *T*_3_ is placed between the highest temperature at which the hydrate phase grows and the lowest temperature at which it melts.

In this work, the initial configuration box is built up, putting in contact the three phases, H_2_ hydrate, aqueous, and pure H_2_. The initial sII H_2_ hydrate phase is built as explained in H–L_w_ Equilibria: Solubility of H_2_ in Liquid Water When It Is in Contact with H_2_ Hydrate section. Particularly, it is formed by a 2 × 2 × 2 unit cell with 1–3 H_2_ occupancy for the hydrate phase in contact with an aqueous phase with 1088 water molecules and with an H_2_ phase with 600 H_2_ molecules. Due to the use of periodic boundary conditions, this arrangement ensures that the three phases are in contact, with one of the phases surrounded by the other two. We choose the 1–3 occupancy since it has been shown that with the most negative values of Δμ_N_^EC^ as a function of the supercooling degree at 185 MPa. As we explained previously, this means that the 1–3 occupancy, from a thermodynamic point of view, is the most favored stoichiometry. Simulations were carried out at 100, 185, and 300 MPa and at different temperatures. As it has been explained in Section II, we propose in this work an extra parameter, *b*_0_, for the Berthelot modified combination rule (see eq 2) proposed by Michalis et al.^18^ in order to improve the predictions of the *T*_3_ values in the 100–300 MPa pressure range. Although the modified factor, χ, proposed by Michalis et al.^18^ gives an excellent prediction of the H_2_ solubility in water and improves the *T*_3_ predictions, the *T*_3_ values obtained still underestimate the experimental results reported in the literature^21,35^ by 5–7 K.

Figure 8 shows the potential energy results obtained at 100, 185, and 300 MPa and at different temperatures. From the results presented in Figure 8 and following the procedure explained previously, it is easy to determine that 263(1), 270(1), and 273(1) K are the *T*_3_ values obtained at 100, 185, and 300 MPa, respectively. As can be seen in Table 4 and Figure 9, the results obtained in this work using the direct coexistence technique and the additional *b*_0_ parameter in eq 2 are equal, within the error bars, to the experimental data reported in the literature.^21,35^ It is also interesting to remark that the result obtained by the solubility method for the 1–3 H_2_ hydrate occupancy is very close to the experimental data, slightly underestimating the experimental *T*_3_ value at the same time that provides an accurate estimation of the H_2_ solubility value in an aqueous phase. On the other hand, the addition of the *b*_0_ parameter in eq 2 improves the prediction of the *T*_3_ values but the H_2_ solubility in an aqueous phase is slightly overestimated.^18^ At this point, it is up to the lector to prioritize the prediction of the *T*_3_ or the H_2_ solubility by taking into account or not the *b*_0_ parameter in eq 2. In both cases, the results obtained by both modified combining rules are excellent.

**Table 4:** Dissociation Temperature of the H_2_ Hydrate Obtained in This Work from the Direct Coexistence Technique (*T*_3_^DC^)­^a^

*P* (MPa)	*T*_3_^DC^ (K)	*T*_3_^EXP^ (K)
100	263(1)	264.1
185	270(1)	269.9/271.6
300	273(1)	274.6

a Results from the experimental data reported in the literature^21,35^ (*T*_3_^EXP^) are also shown.

**Figure 8:**
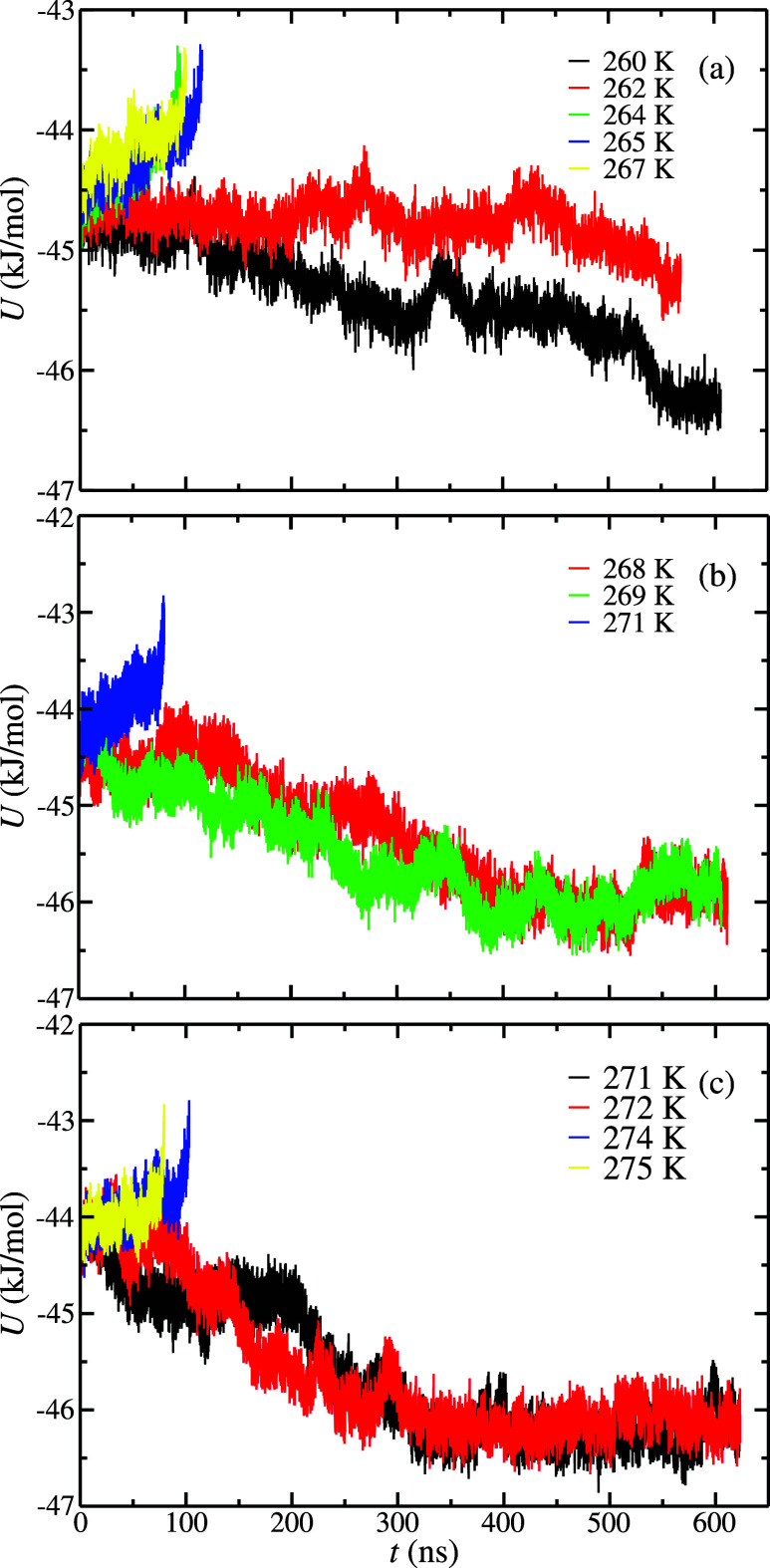
Potential energy as a function of time as obtained from *NPT* molecular dynamic simulations at different temperatures and pressures. (a)–(c) Correspond to results obtained at 100, 185, and 300 MPa, respectively. In all cases, the initial H_2_ hydrate phase contains 1 H_2_ molecules in the small D cages and 3 H_2_ molecules in the large H cages (1–3 occupancy).

**Figure 9:**
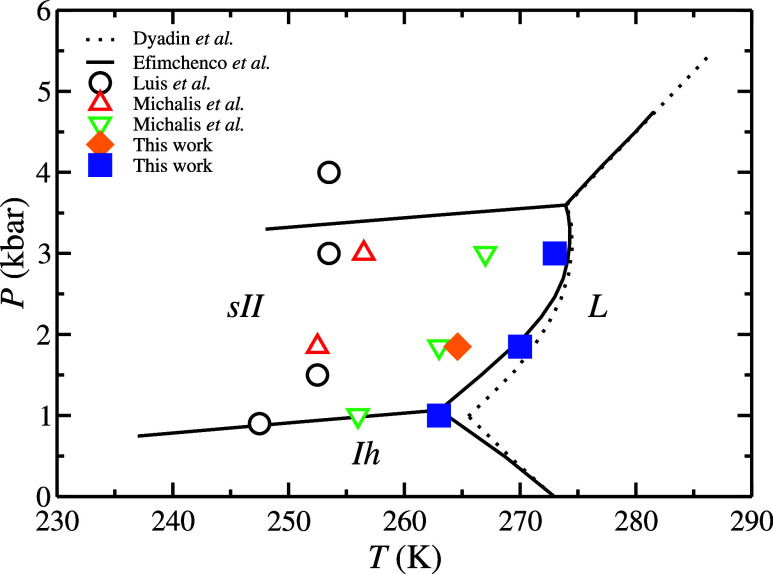
H_2_ hydrate phase diagram. The sII, I*_h_*, and L regions stand for sII H_2_ hydrate, hexagonal ice, and liquid water, respectively. Continuous and dotted black lines correspond to the experimental data reported by Efimchenco et al.^35^ and Dyadin et al.^21^ respectively. Open symbols correspond to the simulation predictions of the three-phase coexistence line reported in the literature by Luis et al.^68^ (black circles) and Michalis et al.^18^ (red up and green down triangles). Finally, filled symbols correspond to the three-phase coexistence conditions obtained in this work. The filled orange represents the predictions obtained by the solubility method at 185 MPa and using the 1–3 occupancy and the original combining rules proposed by Michalis et al.^18^ (i.e., *b*_0_ = 0 in eq 2). Finally, the filled blue squares correspond to the predictions obtained in this work using the direct coexistence technique and the extra parameter *b*_0_ = 0.1 with 1–3 H_2_ hydrate occupancy.

## Conclusions

In this work, we determined the three-phase H–L_w_–L_H_2__ coexistence line following two different molecular computer simulation methods. In both methods, the water and H_2_ molecules are described using the TIP4P/Ice^56^ and a modified version of the SG model^18,29,57^ respectively. In both cases, the Berthelot combination rule for the water-H_2_ interactions has been modified to improve the H_2_ solubility and the *T*_3_ predictions. First, we study the effect of the H_2_ hydrate occupancy on the *T*_3_ value at 185 MPa using the solubility method and 5 different H_2_ hydrate occupancies (1–1, 1–2, 2–2, 1–3, and 1–4). We use the same modification of the Berthelot combining rule proposed by Michalis et al.,^18^ which provides an accurate estimation of the H_2_ solubility in an aqueous phase but slightly underestimates the *T*_3_ value. From this analysis, we conclude that the effect of the H_2_ hydrate occupancy on the *T*_3_ value is negligible since all the occupancies considered in this work provide almost the same *T*_3_ result. The *T*_3_ values obtained following this procedure are in very good agreement with the experimental data reported in the literature^21,35^ although they are slightly underestimated.

We also estimate the driving force for nucleation, Δμ_N_^EC^, of the H_2_ hydrate as a function of the supercooling degree for the five different occupancies considered in this work. We find that multiple occupancy of the large H cages is favored in general, becoming Δμ_N_^EC^ more negative when the occupancy is increased from 1 to 4 H_2_ molecules, reaching the most negative value when the H cages are occupied by 3 H_2_ molecules while the small D cages are single occupied. We also find that the double occupancy of the D and H cages is not favored, becoming Δμ_N_^EC^ more positive than that when both cages are singly occupied. This is due to the high repulsion suffered by both H_2_ molecules encapsulated inside the small D cages. To the best of our knowledge, this is the first time that the effect of the occupancy on the dissociation temperature and the driving force for nucleation of the H_2_ hydrate have been determined from computer simulations.

Finally, we study the dissociation temperature at three different pressures (100, 185, and 300 MPa) by using the direct coexistence technique. We perform the simulations with an initial 1–3 H_2_ hydrate phase and, in order to improve the *T*_3_ predictions, we propose a new modification of the Berthelot combining rule for the water–H_2_ cross-interaction. The results obtained with this new combining rule modification are in excellent agreement with the experimental results reported in the literature.^21,35^

## Data Availability

The data that support the findings of this study are available within the article.
